# Updated aspects of alpha‐Solanine as a potential anticancer agent: Mechanistic insights and future directions

**DOI:** 10.1002/fsn3.4221

**Published:** 2024-08-29

**Authors:** Sudeshna Nandi, Rimpa Sikder, Anish Nag, Somanjana Khatua, Surjit Sen, Nilanjan Chakraborty, Arghya Naskar, Kairat Zhakipbekov, Krishnendu Acharya, Solomon Habtemariam, Dilek Arslan Ateşşahin, Tamar Goloshvili, Afaf Ahmed Aldahish, Javad Sharifi‐Rad, Daniela Calina

**Affiliations:** ^1^ Molecular and Applied Mycology and Plant Pathology Laboratory, Department of Botany University of Calcutta Kolkata India; ^2^ Department of Life Sciences CHRIST (Deemed to be University) Bangalore Karnataka India; ^3^ Department of Botany, Faculty of Science University of Allahabad Prayagraj Uttar Pradesh India; ^4^ Department of Botany Fakir Chand College Kolkata India; ^5^ Department of Botany Scottish Church College Kolkata India; ^6^ Department of Organization and Management and Economics of Pharmacy and Clinical Pharmacy Asfendiyarov Kazakh National Medical University Almaty Kazakhstan; ^7^ Pharmacognosy Research & Herbal Analysis Services UK Kent UK; ^8^ Department of Plant and Animal Production, Baskil Vocational School Fırat University Elazıg Turkey; ^9^ Department of Plant Physiology and Genetic Resources Institute of Botany, Ilia State University Tbilisi Georgia; ^10^ Department of Pharmacology, College of Pharmacy King Khalid University Abha Kingdom of Saudi Arabia; ^11^ Department of Biomedical Sciences College of Medicine, Korea University Seoul Republic of Korea; ^12^ Department of Clinical Pharmacy University of Medicine and Pharmacy of Craiova Craiova Romania

**Keywords:** anticancer mechanisms, apoptosis, glycoalkaloids, *Solanaceae*, α‐Solanine

## Abstract

Cancer remains a critical global health challenge, with limited progress in reducing mortality despite advancements in diagnosis and treatment. The growing resistance of tumors to existing chemotherapy exacerbates this burden. In response, the search for new anticancer compounds from plants has intensified, given their historical success in yielding effective treatments. This review focuses on α‐solanine, a glycoalkaloid primarily derived from potato tubers and nightshade family plants, recognized for its diverse biological activities, including anti‐allergic, antipyretic, anti‐inflammatory, anti‐diabetic, and antibiotic properties. Recently, α‐solanine has gained attention as a potential anticancer agent. Utilizing resources like PubMed/MedLine, ScienceDirect, Web of Science, Scopus, the American Chemical Society, Google Scholar, Springer Link, Wiley, and various commercial websites, this review consolidates two decades of research on α‐solanine's anticancer effects and mechanisms against nine different cancers, highlighting its role in modulating various signaling pathways. It also discusses α‐solanine's potential as a lead compound in cancer therapy. The abundant availability of potato peel, often discarded as waste or sold cheaply, is suggested as a sustainable source for large‐scale α‐solanine extraction. The study concludes that α‐solanine holds promise as a standalone or adjunctive cancer treatment. However, further research is necessary to optimize this lead compound and mitigate its toxicity through various strategies.

## Graphical abstract

α‐Solanine, extracted from vegetables has a role as an anticancer compound. Illustrated are its molecular structure and experimental evidence of anticancer action in cancer cell lines and animal models, demonstrating increased apoptosis, cell cycle arrest, metastasis reduction and angiogenesis inhibition.

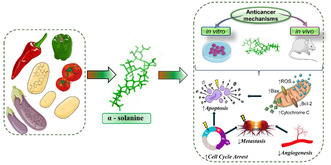

## 1 INTRODUCTION

Cancer is one of the most common human diseases and a leading cause of death globally (GBD 2019 Colorectal Cancer Collaborators, 2022; GBD 2019 Lip, Oral, and Pharyngeal Cancer Collaborators, et al., 2023). In 2022, approximately 19 million new cases of cancer and 12 million cancer deaths were registered (Siegel et al., 2022). Over the next 20 years, the number of cancer incidences is expected to elevate to more than 28 million, and these estimates might help in planning better prevention measures and therapeutic strategies (Sung et al., 2021). Among the various factors that affect susceptibility to cancer are genetic predisposition, virus infection, lifestyle, and chronic inflammation (Rahaman et al., 2023) Over the last few decades, both the diagnosis and medication of malignant tumors have been extensively studied. The hope is that investments in the initial detection, medication, and more targeted cancer control interventions would reduce the current level of cancer mortality (GBD 2019 Lip, Oral, and Pharyngeal Cancer Collaborators, 2023; Loud & Murphy, 2017). For those people who are at high risk of cancer incidence, the chemoprevention strategy could be employed to delay or prevent carcinogenesis. Unlike the chemotherapeutic agents which have been employed with known clinical efficacy for such a long time, the effectiveness of chemopreventive agents and safety to human populations are yet to be established. Concerning the search for new strategies for treating cancer, the role of global plant resources with millions of chemical entities must be emphasized (Ozkan et al., 2023; Prasher et al., 2023). Through millions of years of evolution, plants have been proven to provide a huge quantity of biologically active substances that target human diseases (Chaudhary et al., 2023; Zahra et al., 2023). Hence, modern medicine employs a significant number of plant‐derived natural products for the prevention and treatment of several ailments; in this regard, several anticancer therapies are enforced either as monotherapy or in combination with conventional medicine (Bao et al., 2022). All these strategies aim to inhibit proliferation, promote apoptosis, interfere with invasion and metastasis, and inhibit deregulated survival signaling pathways in the cancer cell. The systematic search for potential anticancer therapeutic agents traditionally comes from studies on the use of plants by indigenous people of various cultures. Some of the classical anticancer agents still in use today, including podophyllotoxin and derivatives and the vinca alkaloids (vinblastine and vincristine), result from the ethnobotanical approach to drug discovery (Banyal et al., 2023). The utilization of medicinal plants belonging to the *Solanaceae* family for cancer treatment has also been well‐established (Khan et al., 2021). *Solanaceae* plants are rich in bioactive metabolites, including glycosides, alkaloids, and lignans (Afroz et al., 2020). The two major bioactive phytochemicals are, however, alkaloids (Jayakumar & Murugan, 2016) or glycoalkaloids (GAs), which are known to prevent the growth of several cancer cells, including the liver, skin, breast, prostate, and colon. Plants manufacture these glycoalkaloids as natural toxins for shielding from raucous environmental conditions like phytopathogen attacks, extreme cold, and vertebrate feeding (Friedman, 2006). Hence, a large number of scholars focussed their antitumor potential studies on α‐solanine. The structure of α‐solanine resembles human steroidal hormones like androgens, progesterone, estrogen, and other sex hormones (Pan et al., 2016). α‐Solanine has been shown to display anti‐inflammatory, antipyretic, anti‐allergic, anti‐diabetic, and antibiotic activity against viruses, bacteria, protozoa, and fungi (Hassan et al., 2021). To date, several in vitro and in vivo studies have shown its antiproliferative activity against cancer (Luo et al., 2021).

*Solanum tuberosum* L., traditionally valued for its medicinal properties, is a key source of glycoalkaloids. Through detailed purification and analysis, it was determined that solanine, a natural glycoalkaloid found in *S. tuberosum* L., stands out as a critical bioactive component (Winkiel et al., 2022). Since being discovered in 1820, solanine has been identified in various plants, including potatoes and tomatoes. The term “solanine” encompasses α‐solanine, β‐solanine, and γ‐solanine, with α‐solanine being the predominant form in *S. tuberosum* (Friedman, 2015). This has led numerous researchers to concentrate on the antitumor potential of α‐solanine. Structurally, α‐solanine bears resemblance to human steroidal hormones such as androgens, progesterone, estrogen, and other sex hormones (Pan et al., 2016). α‐Solanine is known for its diverse biological activities, including anti‐inflammatory, antipyretic, anti‐allergic, and anti‐diabetic properties, and its antibiotic effectiveness against viruses, bacteria, protozoa, and fungi (Hassan et al., 2021). Numerous in vitro and in vivo studies to date have demonstrated its anti‐proliferative effects against cancer. This review synthesizes recent research findings regarding the anticancer properties of α‐solanine, suggesting that it holds significant promise as a potential therapeutic agent.

## 2 REVIEW METHODOLOGY

The selection of studies for this comprehensive review was guided by specific inclusion and exclusion criteria designed to focus on high‐quality, relevant, and recent research on the pharmacological properties of α‐solanine as an anticancer agent.

Inclusion Criteria: studies published between 2000 and 2022; research articles were retrieved from widely recognized databases and search engines, including PubMed/MedLine, ScienceDirect, Web of Science, Scopus, the American Chemical Society, Google Scholar, Springer Link, and Wiley. Search Terms: the search was conducted using the following MeSH terms: “Neoplasms/drug therapy”, “Signal Transduction”, “Solanine/pharmacology”, “Solanum tuberosum”, “Solanine/therapeutic use”, “Cell Line, Tumor”, “Animals”, “Xenograft Model Antitumor Assays/methods”, “Antineoplastic Agents /pharmacology”, and “Antineoplastic agents/therapeutic use”; only publications in the English language were considered; publications were selected based on their innovativeness, clarity of explanation, systematic nature, and impact within the field; preference was given to studies that provided clear methodological details, including experimental conditions and comparative analyses.

Exclusion Criteria: articles focusing on the effects of crude plant mixtures or homeopathic preparations of Solanum species were excluded to maintain the focus on α‐solanine; studies published in languages other than English were excluded due to language constraints; publications that did not directly address the pharmacological effects of α‐solanine or were deemed outdated (published before 2000) were not included in this review; studies from non‐peer‐reviewed sources or those lacking in scientific rigor and clarity were excluded.

The search strategy involved an approach to identifying relevant literature. The process included the following: initial search – utilizing the specified databases and search engines with the defined MeSH terms to gather a broad range of potentially relevant articles; titles and abstracts were initially screened to assess relevance based on the inclusion and exclusion criteria; full texts of potentially relevant articles were obtained and thoroughly reviewed for detailed assessment against the inclusion criteria; and a total of 94 publications were ultimately selected based on their alignment with the inclusion criteria and their contribution to understanding the pharmacological effects of α‐solanine in cancer therapy. To ensure the high standard of the included studies, a quality assessment was conducted. This assessment focused on the study design, methodology, and clarity and consistency of the results and conclusions. This methodology ensured a comprehensive, systematic, and critical review of the available literature on α‐solanine as a prospective anticancer agent, providing a solid foundation for the insights and future directions discussed in this review. The scientific name of the plant species has been validated according to World Flora Online (WFO, 2021) and the chemical structures according to PubChem (PubChem, 2022). The most representative pharmacological data are summarized in tables and figures.

## 3 SOURCES AND PHYTOCHEMISTY OF α‐SOLANINE

### 3.1 Source of α‐solanine

A range of antimicrobial and anti‐cancer agents of plant origin have been evaluated under clinical conditions (Harvey et al., 2015; Jiang et al., 2016). Most of these therapeutically useful plants show activity due to their phytochemicals, mainly polyphenols and alkaloids (Jayakumar & Murugan, 2016). As indicated in the previous section, the Solanaceae plant also synthesizes glycoalkaloids (GAs) as natural toxins, which also prevent the growth of many cancer cells. The potato, *S. tuberosum* L. and other nightshade plants contain steroidal glycoalkaloids commonly called solanines, of which α‐solanine accounts for about 95% of the total GAs (Ji & Gao, 2012).

α‐Solanine is found in specific tissues of the potato plant, including leaves, shoots, stems, blossoms, tubers, eyes, green peels, and sprouts, with notably higher concentrations observed in green peels and sprouts (Maga & Fitzpatrick, 1980; Meng et al., 2016). Research indicates that α‐solanine synthesis increases on the cut surfaces of potatoes, and factors such as mechanical damage and aging can further promote its production (Allen, 1968; Beier, 1990; Sinden, 1972). Additionally, exposure to light, either in the field or in market settings, enhances the synthesis of this compound in potato tubers (Beier, 1990). Potatoes that turn green from light exposure are often deemed unsuitable for consumption due to elevated α‐solanine levels (Morris, 1984). Besides the potato, α‐solanine is also found in other members of the Solanaceae family, including *S. nigrum*, *S. villosum*, *S. elaeagnifolium*, *S. melongena*, *S. lycopersicum*, and *Capsicum annuum* (Sammani et al., 2013). This compound has been detected in plants outside the nightshade family as well, such as *Malus domestica* from the Rosaceae family and *Beta vulgaris* from the Amaranthaceae family.

The presence of α‐solanine in some nightshade plants and different potato products is presented in Table 1. Potatoes are considered as the primary staple crop that people consume globally, and potato peel is a potential source of α‐solanine that can be extracted in a sustainable manner from the global utilization of this plant. This GA can also be sourced from effluents from potato starch factories and in the potato waste products produced during potato processing (Bushway et al., 1984).

**TABLE 1:** Plant sources of α‐solanine.

Common name	Scientific name and family	Parts	Concentration	Reference
Potato	*Solanum tuberosum* (Solanaceae)	Peel	273 μg/g	Hossain et al. (2014)
		Mashed Potato Flakes	15–28 mg/kg	Kondamudi et al. (2017)
		Tuber	6.5 mg/kg	Kondamudi et al. (2017)
		Potato Chips	2–13 mg/kg	Alves‐Filho et al. (2018)
Hairy nightshade	*S. villosum* (Solanaceae)	Fruit and leaves	—	Sammani et al. (2013)
Tomato	*S. lycopersicum* (Solanaceae)	Fruits (green), flowers, leaves	0.05 mg/g fruit	Kozukue et al. (2004)
Ground cherries	*Physalis peruviana* (Solanaceae)	Immature fruit	—	Singh et al. (2019)

### 3.2 Chemistry of α‐solanine

**FIGURE 1:**
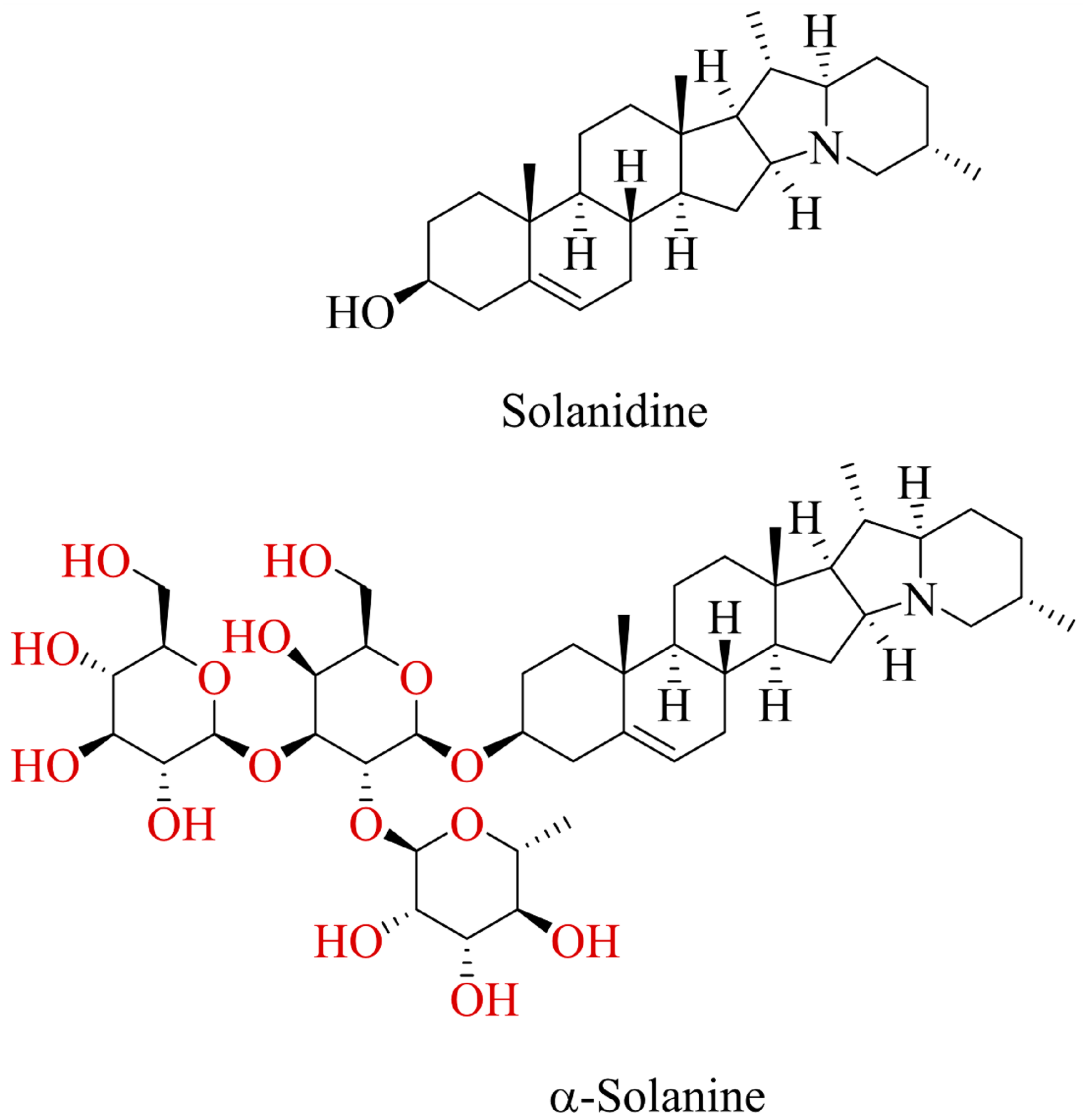
The chemical structure of α‐solanine and its aglycone solanidine.

α‐Solanine, as a steroidal glycoalkaloid, is a heterocyclic base containing a nitrogen atom and a sugar moiety attached at the 3‐OH position of the steroidal skeleton (Figure 1). In general, the steroidal glycoalkaloids contain a combination of monosaccharide sugars like D‐glucose, D‐galactose, D‐xylose, and/or L‐rhamnose (Friedman, 2004; Nepal & Stine, 2019). The molecular formula of α‐solanine is C_45_H_73_NO_15_. It is a trisaccharide derivative of solanidine, with a molecular weight of 868.1 g/mol and a precursor mass of 868.5052971 in the LC/MS–MS analysis (https://pubchem.ncbi.nlm.nih.gov/).

### 3.3 Biosynthesis of α‐solanine

**FIGURE 2:**
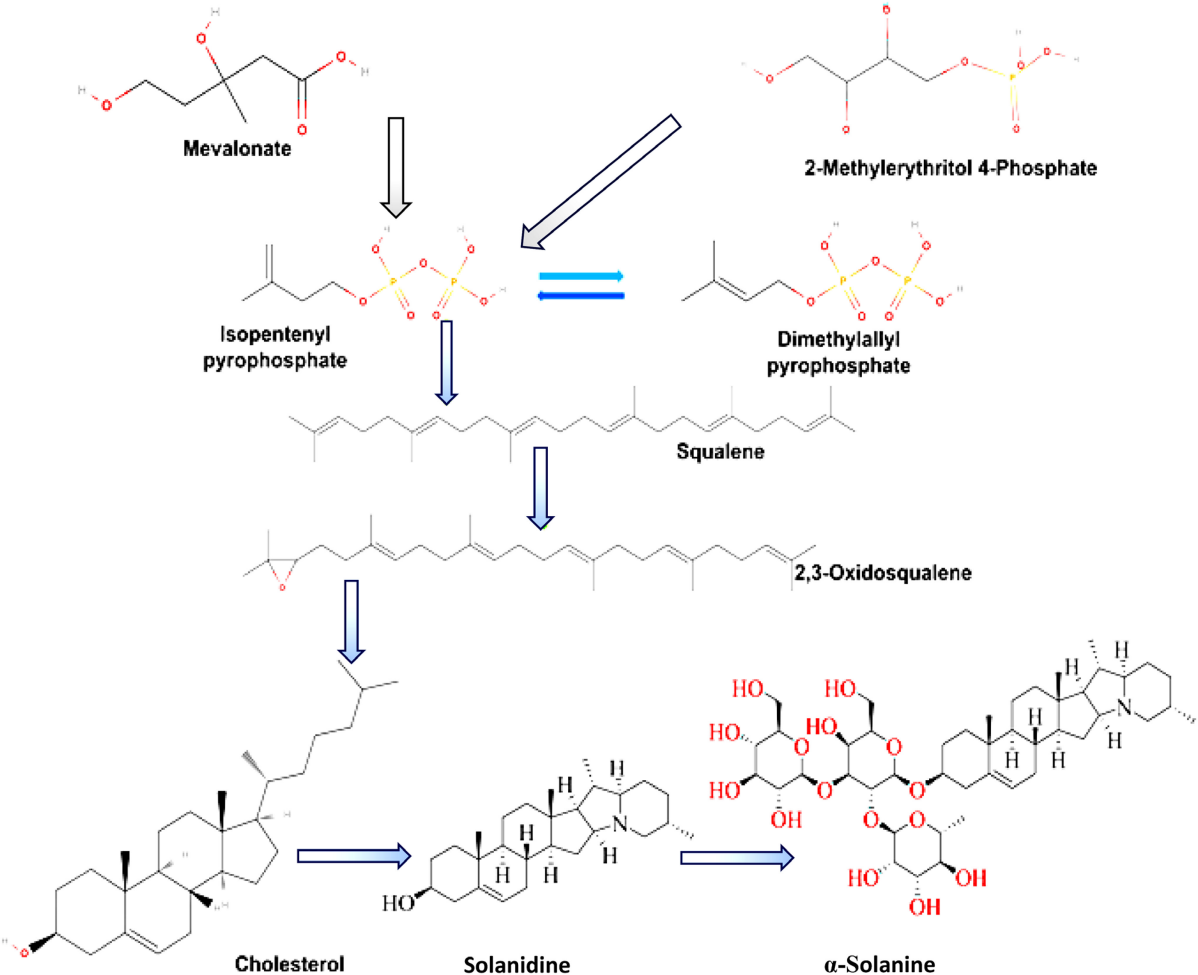
Biosynthetic Pathway of α‐Solanine. The figure illustrates the metabolic pathway leading to the biosynthesis of α‐solanine, starting from the fundamental precursor, mevalonate. The pathway progresses through several intermediates, including isopentenyl pyrophosphate, dimethylallyl pyrophosphate, and squalene, eventually forming 2,3‐oxidosqualene. This compound is then converted into cholesterol, which serves as a substrate for the synthesis of solanidine. Solanidine is subsequently glycosylated to form α‐solanine. The blue arrows indicate the enzymatic steps involved in the transition from cholesterol to solanidine and finally to α‐solanine, highlighting the intricate network of reactions within the sterol metabolic pathway; this process is integral to the production of glycoalkaloids, which are significant for their roles in plant defense and potential therapeutic applications.

Two major biosynthesis pathways of α‐solanine were reported in the literature, namely the mevalonate (MEV) and methylerythritol phosphate (MEP) pathways in the cytosolic compartment and plastids, respectively (Figure 2). Studies further showed two C5 or isoprene building blocks of terpenoids, isopentenyl pyrophosphate (IPP) and dimethylallyl pyrophosphate (DMAPP), as the precursors for both pathways (Bach, 1995; Eisenreich et al., 2004; Heftmann, 1983; Kuzuyama & Seto, 2012; Lichtenthaler et al., 1997). In an early study, Guseva et al. (1962) reported the involvement of the acetate and mevalonate biosynthesis pathways in the production of α‐solanine in potato leaves, seeds, and sprouts (Guseva et al., 1962; Kozukue et al., 2001). Furthermore, the exchange of IPP and DMAPP precursors between the plant compartments indicated the role of MEP, as the secondary route of synthesis for the steroidal glycoalkaloids (Hampel et al., 2005; Laule et al., 2003; Schramek et al., 2010). As with triterpene synthesis in plants, the biosynthesis of glycoalkaloids followed a series of reactions, including the condensation of two isoprene units to make geranyl pyrophosphate, followed by the addition of another isoprene unit, leading to the formation of the C15 terpene precursor, farnesyl pyrophosphate. The condensation of two farnesyl pyrophosphates leads to the formation of the C30 compound squalene (tail‐to‐tail condensation of two C15 units). Through multiple intermediates (2,3‐oxidosqualene; lanosterol/cycloartenol), squalene is converted into C27 precursor compounds (e.g. cholesterol). At the later stage of the biosynthesis, C27 precursors incorporate hydroxylation at multiple positions, namely C‐16, C‐22, and C‐26, leading to the formation of aldehyde intermediates. Finally, 25*S* solanidine is formed via a series of reactions, like the synthesis of the F‐ring of solanidine (C27) via 25*S* teinemine (C27) and etioline (C27), and dihydroxylation and ring closure at position C‐16. At the end, α‐solanine is formed through the glycosylation of 25*S* solanidine and the catalysis of solanidine glycosyl transferases (Bergenstråhle et al., 1992; Cárdenas et al., 2015; Heftmann, 1983; Kaneko et al., 1976; McCue et al., 2006; Nes, 2011; Suzuki & Muranaka, 2007). Recently, (2021) confirmed the mevalonate‐dependent biosynthesis of the IPP and the involvement of DMAPP through ^13^C radiolabelling echnique. Further, the cryslallization of the 2,3‐oxidosqualene precursor into the solanidine aglycone backbone involving a non‐stereo selective hydroxylation step was also suggested.

### 3.4 Natural derivatives of α‐solanine

**FIGURE 3:**
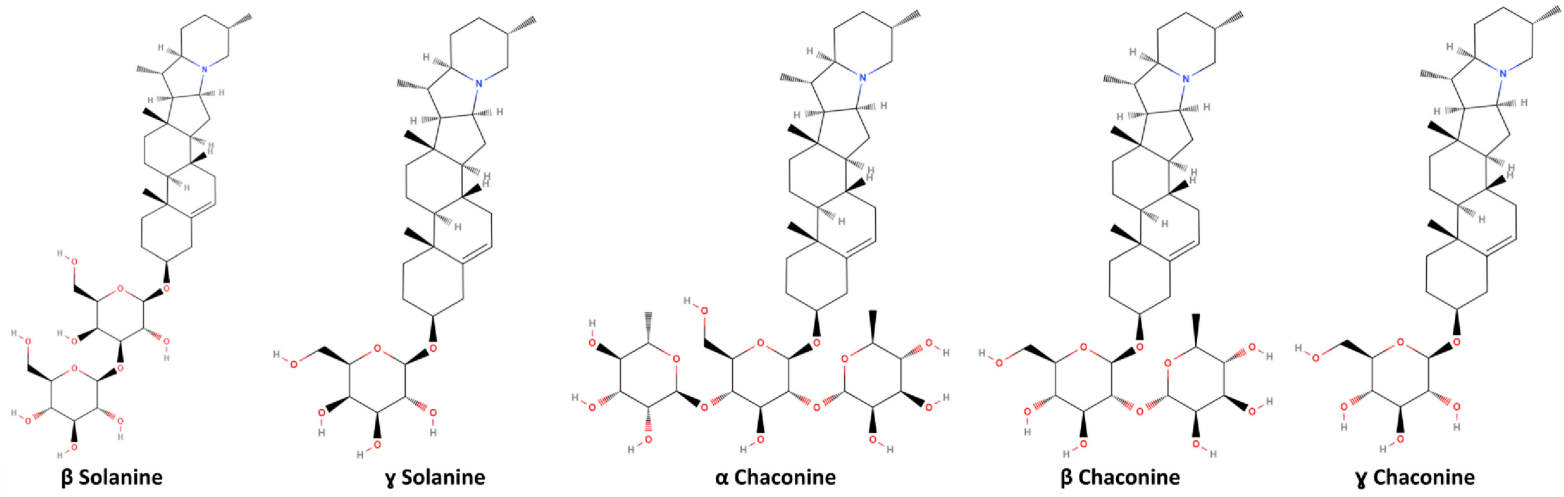
Two‐dimensional structures of natural derivatives of α‐solanine.

α‐Solanine is a trisaccharide saponin, containing three sugar moieties, namely, D‐gluocse, L‐rhamnose, and D‐galactose. There are various natural derivatives of these compounds, varying with the number and position of sugar moieties, such as β‐solanine, ɣ‐solanine, α‐chaconine, β‐chaconine, and ɣ‐chacoanine (Alves‐Filho et al., 2018; Baur et al., 2021; Zimowski, 1997). The structures of the natural derivatives of α‐solanine are shown in Figure 3.

## 4 PHARMACOKINETICS DATA

α‐Solanine is a glycoalkaloid predominantly found in species of the nightshade family, notably in potatoes and tomatoes. It has drawn scientific interest due to its potential therapeutic applications and toxicity concerns; understanding the pharmacokinetics and appropriate dosing of α‐solanine is crucial for its safe and effective use (Hassan et al., 2021). α‐Solanine is absorbed through the gastrointestinal tract following oral administration; the compound's bioavailability may be influenced by various factors, including the presence of food, gastric pH, and intestinal metabolism (Abuhelwa et al., 2017). Studies indicate a variable absorption rate, with peak plasma concentrations typically observed within 1–2 h post‐ingestion (EFSA Panel on Contaminants in the Food Chain (CONTAM) et al., 2020). Once absorbed, α‐solanine is widely distributed throughout the body; it has a high affinity for cell membranes due to its lipophilic nature. α‐Solanine undergoes hepatic metabolism; the primary metabolic pathways involve hydrolysis of the glycosidic bond, leading to the formation of solanidine and sugars; the exact enzymes involved in its metabolism have not been fully elucidated, but Phase I and Phase II metabolic reactions are believed to play roles (EFSA Panel on Contaminants in the Food Chain (CONTAM) et al., 2020). The excretion of α‐solanine is primarily through the kidneys, with both the parent compound and its metabolites being detectable in urine; the elimination half‐life of α‐solanine is variable but generally ranges from 10 to 20 h, indicating a moderate rate of clearance from the body (Kasimir et al., 2023). In an experimental study, the pharmacokinetics of α‐solanine were assessed in rats and hamsters, given both orally at 170 μg/kg and intravenously at 54 μg/kg, and the findings revealed that after intravenous administration, both species displayed similar blood toxicokinetics for total radioactivity, but rats showed a more efficient plasma clearance. Notably, systemic and metabolic clearance rates of alpha‐solanine in rats were approximately 1.6 and 2.7 times higher, respectively, than in hamsters. When administered orally, total radioactivity exhibited a bioavailability of about 29% in rats and 57% in hamsters, while the bioavailability of unchanged alpha‐solanine was significantly lower at 1.6% in rats and 3.2% in hamsters compared to the intravenous route. The elimination half‐life of alpha‐solanine post‐oral administration was four times shorter in rats and two times shorter in hamsters than after intravenous administration. Moreover, a marked retention of radioactivity was observed in hamsters, with only 40% of the oral dose excreted within 7 days, in contrast to 90% in rats. Based on these pharmacokinetic and toxicokinetic differences, hamsters were concluded to be a more suitable model than rats for (sub)‐chronic toxicity studies of α‐solanine (Groen et al., 1993).

## 5 EFFECTS OF FOOD PROCESSING ON α‐SOLANINE LEVELS AND BIOAVAILABILITY

α‐Solanine, a natural glycoalkaloid found predominantly in potatoes, can exhibit both toxic and beneficial health effects, depending on the dose. Food processing methods, such as boiling, frying, and baking, significantly influence the concentration of α‐solanine, thereby affecting its bioavailability and potential health impacts (Urugo & Tringo, 2023).

Boiling is a common method of cooking potatoes that involves immersing them in water at high temperatures (Buratti et al., 2020). This process can lead to the leaching of α‐solanine into the cooking water, resulting in a significant reduction of its levels in the potato flesh. A study found that 93.9% of α‐chaconine and 95.9% of solanine remained in potatoes after boiling, indicating that boiling may not be as effective as believed in reducing these alkaloids. The study also explored other cooking methods, finding microwaving and deep‐frying at certain temperatures to offer some reduction (Takagi et al., 1990). Another research evaluated new strategies for reducing α‐solanine and α‐chaconine in potatoes, achieving a reduction of 43% for α‐solanine and 27% for α‐chaconine under specific conditions, though not specifically through boiling (Romanucci et al., 2018). An investigation into the effects of peeling and cooking methods on potatoes found that boiling reduced the contents of total glycoalkaloids, including α‐chaconine and α‐solanine, making it a favorable cooking method for reducing these compounds (Lachman et al., 2013).

Frying potatoes, either by deep‐frying or pan‐frying, subjects them to high temperatures, which can degrade α‐solanine. The reduction in solanine levels during frying is attributed to thermal degradation and leaching into the frying oil. However, the extent of degradation varies based on the frying temperature, time, and thickness of the potato slices. Thinly sliced potatoes, such as chips or fries, may experience a greater reduction in solanine content due to the increased surface area exposed to the oil and high temperatures.

Baking involves cooking potatoes in an oven at high temperatures without immersing them in water or oil (Buratti et al., 2020). While baking can also lead to a decrease in α‐solanine levels, the reduction is generally less significant compared to boiling or frying. The solanine reduction during baking is primarily due to thermal degradation, and factors such as baking temperature, duration, and the potato's size can influence the extent of solanine reduction (D'Amelia et al., 2022).

Microwaving potatoes is another quick method of cooking that can affect solanine levels; similar to baking, microwaving can lead to a reduction in solanine content due to heat exposure (Buratti et al., 2020). However, the reduction might not be as significant as in boiling, and it can vary widely depending on the microwave's power and the cooking time (D'Amelia et al., 2022). Peeling potatoes can also reduce α‐solanine content since solanine is more concentrated in the skin and the outer layers of the flesh. Removing the skin and a thin layer of the flesh can effectively decrease the solanine levels in the final dish (Hassan et al., 2021).

The reduction of α‐solanine levels through various food processing methods can minimize the risk of solanine toxicity, which can cause symptoms such as nausea, vomiting, and neurological disturbances (Delbrouck et al., 2023); and it is essential to balance the reduction of potentially harmful compounds like solanine with the preservation of beneficial nutrients because some processing methods, particularly those involving high temperatures and water, can also lead to the loss of vitamins and minerals (Levaj et al., 2023).

In conclusion, food processing methods can significantly impact the levels and bioavailability of α‐solanine in potatoes, with boiling and frying offering more substantial reductions compared to baking and microwaving. Understanding these effects is important for both food safety and nutritional optimization, allowing consumers and food manufacturers to make informed decisions about potato preparation methods to minimize health risks associated with solanine while preserving the nutritional value of potatoes.

## 6 ANTICANCER EFFECTS OF α‐SOLANINE: UNDERLYING CELLULAR AND MOLECULAR MECHANISMS

**FIGURE 4:**
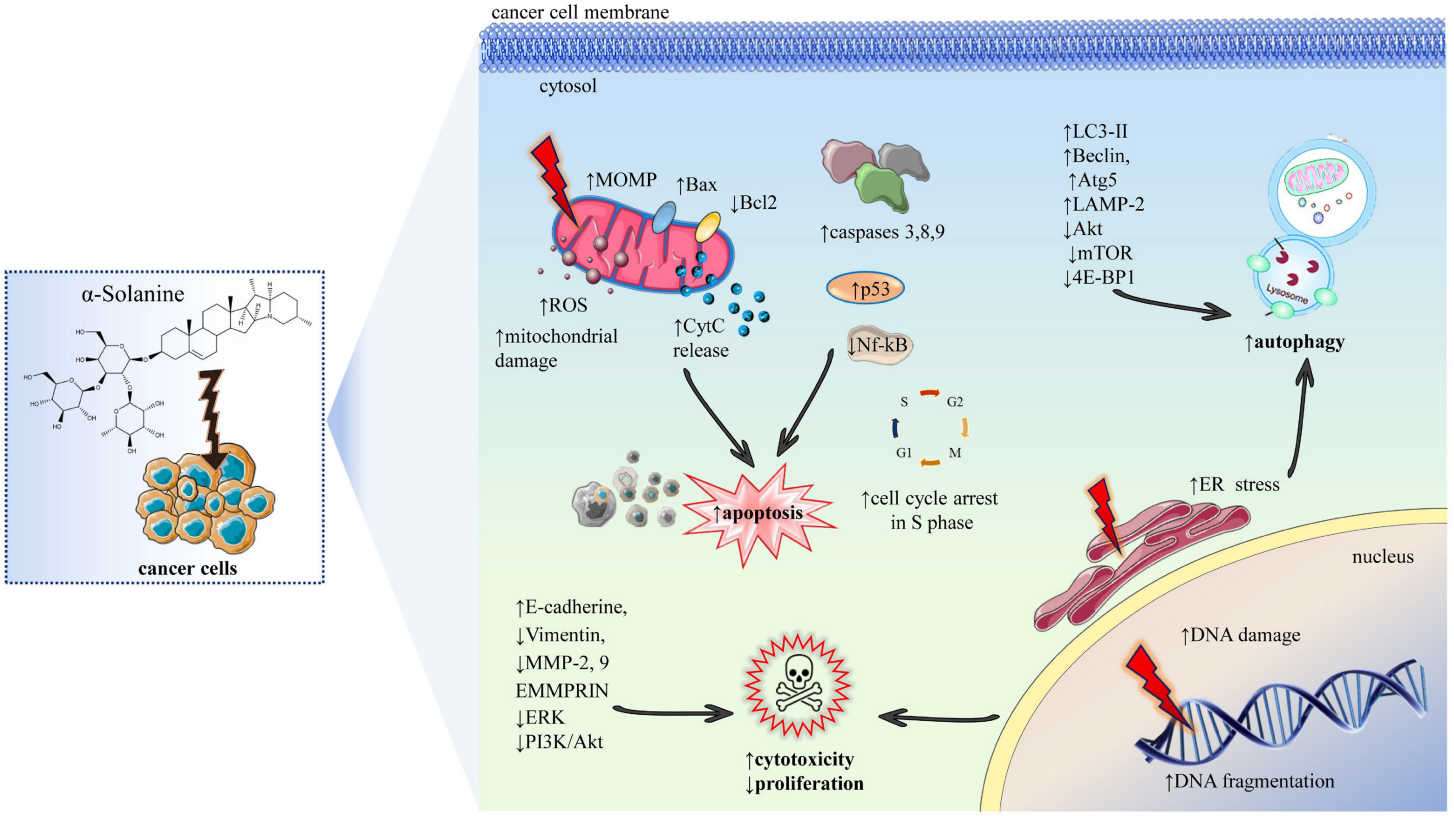
Mechanistic pathways of α‐solanine‐induced apoptosis and anticancer activity. The figure depicts the multiple cellular and molecular mechanisms by which α‐solanine exerts its anticancer effects. It illustrates the pathways leading to apoptosis, such as mitochondrial dysfunction, modulation of apoptosis‐related proteins, cell cycle arrest, and DNA damage. Additionally, it demonstrates the role of α‐solanine in inducing autophagy and ER stress and its impact on signaling pathways that govern cell proliferation and metastasis. ↑, increase; ↓, decrease; 4E‐BP1, 4E‐Binding Protein 1; Akt, Protein Kinase B; Atg5, Autophagy‐Related 5; Bax, BCL2‐Associated X, Apoptosis Regulator; Bcl2, B‐Cell Lymphoma 2; CytC, Cytochrome C; E‐cadherine, epithelial cadherin; EMMPRIN, Extracellular Matrix Metalloproteinase Inducer; ER stress, Endoplasmic Reticulum Stress; ERK, Extracellular Signal‐Regulated Kinases; GAS5, Growth Arrest‐Specific 5; LAMP‐2, Lysosomal‐Associated Membrane Protein 2; LC3‐II, Microtubule‐Associated Proteins 1A/1B Light Chain 3B; MMP‐2, 9, Matrix Metallopeptidase 2 and 9; MOMP, Mitochondrial Outer Membrane Permeabilization; mTOR, Mammalian Target of Rapamycin; Nf‐kB, Nuclear Factor Kappa B; p53, Tumor Protein p53; PI3K/Akt, Phosphoinositide 3‐Kinases/Protein Kinase B; ROS, Reactive Oxygen Species.

Studies have demonstrated that α‐solanine is one of the most dominant bioactive components of the Solanaceae family (Elizalde‐Romero et al., 2021). Numerous studies also reported that α‐solanine is the main steroidal glycoalkaloid in these plants' anticancer properties. Literature studies stated that α‐solanine exhibited cytotoxicity against multiple cancer cell lines through several mechanisms, including apoptosis induction via different signaling pathways, cell cycle arrest, autophagy, metastasis inhibition, and angiogenesis (Hassan et al., 2021; Lee et al., 2004; Luo et al., 2021). Tables 1 and 2 summarize the anticancer effect of α‐solanine against 9 different types of cancer, both in vitro and in vivo, through several mechanisms, including apoptosis induction, autophagy, cell cycle arrest, angiogenesis, and metastasis inhibition, as also shown in Figure 4.

**TABLE 2:** The molecular targets of α‐solanine against different types of cancer: evidence from preclinical pharmacological studies.

Type of studies					Reference
In vitro studies using cell lines					
Cancer type	Cell lines model/	IC_50_/Working dose	Mechanisms	Primary outcomes	
Pancreatic	SW1990 Panc‐1	5–50 μg/mL	↑p53, ↑Bax, ↓Bcl‐2, ↑Caspase 3, ↓MMP‐2, ‐9	↑Apoptosis ↓Migration	Sun et al. (2014)
	PANC‐1 SW1990 MIA PaCa‐2	3,6, and 9 μg/mL	↓VEGF, ↓CD44, ↓MMP‐2/9, ↓eNOS, ↓EMMPRIN, ↓E‐cadherin ↓Akt/mTOR, ↓Wnt/β‐catenin ↓JAK/STAT	↓Angiogenesis ↓Migration	Lv et al. (2014)
	PC‐3	4, 8, and 12 μM	↑MMP, ↑E‐cadherine, ↓Vimentin, ↓MMP‐2, 9, EMMPRIN, ↓ERK, ↓PI3K/Akt	↓Migration	Shen et al. (2014)
	PANC‐1, BXPC‐3 PATU‐8988	6 μg/μL	↓VEGF, ↓E‐cadherin, ↓p‐ERK1/2, ↓HIF‐1α, ↓STAT3	↓Angiogenesis	Wen et al. (2016)
Esophageal	EC9706 Eca109	20, 40, and 60 μg/mL	↑apoptotic rate, ↓MMP‐2, ‐9, ↑E‐cadherine	↑Apoptosis ↓Migration	Wang, Wu, et al. (2016)
Melanoma	A2058	9.2, 13.8, and 18.4 μM	↓MMP‐2, ‐9	↓Metastasis	Lu et al. (2010)
Non‐small cell lung cancer (NSCLC)	A549 PC‐9	12, 18, and 24 μg/mL IC_50_ = 18 μg/mL	↓GPI, ↓ALDOA, ↓TPI1, ↓PKLR, ↓LDHA, ↓ALDH3	↑Apoptosis ↓Migration	Zou et al. (2022)
	A549 H1299	3 or 6 μmol/L	↓miR‐138, ↓FCK	↓Migration ↓Invasion	Zhang et al. (2016)
	A549	10 μM	↑Oxidative damage, ↑LC3‐II, ↑Beclin, ↑Atg5, ↑LAMP‐2, ↓Akt, ↓mTOR, ↓4E‐BP1, ↑ER stress protein expression	↑Autophagy	Hasanain et al. (2015)
Liver	Hep G2	—	↓miRNAs ↑NF‐κB	↑Apoptosis	Gouhar et al. (2022)
		10 μM	↓Vimentin ↓E‐cadherin, ↓ miR‐21	↓Migration	Lin et al. (2020)
		0.5 mmol	↑ROS, ↑ASK1, ↑TBP‐2, ↓HDAC1	↑Apoptosis	Meng et al. (2016)
		IC_50_ = 14.47 μg/mL	↓Bcl‐2, ↑cell cycle arrest at S phase	↑Apoptosis	Ji et al. (2008)
		0.08; 0.4; 2 μg/mL	↑MMP disruption, ↑Ca^2+^ concentration	↑Apoptosis	Gao et al. (2006)
Prostate	DU145	10–160 μmol IC_50_ = 32.18 μmol	↓Cyclin D1, ↓Cyclin E1, ↓CDK2, ↓CDK4, ↓CDK6, ↓p21, ↑ROS, ↑p38 MAP kinase	↑Apoptosis	Pan et al. (2016)
	PC‐3 and DU145	4–16 μM	↑DNA damage, ↑Apoptotic rate, ↑GAS5 ↓miR18a	↑Apoptosis	Yang et al. (2019)
Colorectal	RKO	19–25 μM IC_50_ = 20.84 μM	↑ROS, ↑Cell cycle arrest at G0/G1 phase, ↑CDK4, ↑ CCND1, ↑Caspase‐3, ‐8, ‐9, ↓MMP‐2, ‐9	↑Apoptosis ↓Migration ↓Invasion	Yan et al. (2020)
	HCT‐116	11–28 μM IC_50_ = 20.32 μM	↑Caspases‐3, ‐8, ‐9		
	SW480, SW620 HT‐29	—	↑Cell cycle arrest ↓S100P protein	↑Apoptosis ↓Migration ↓Invasion	Ni et al. (2018)
Choriocarcinoma	JEG‐3	30 μM	↓MMP‐2, ↓MMP‐9	↓Migration ↓Invasion	Ni et al. (2018)
Leukemia	AML‐193	5, 10, and 20 μM IC_50_ = 10 μM	↑Bax, ↑miR‐16, ↓Bcl‐2	↑Apoptosis	Zheng et al. (2020)

In vivo studies using animal models					Reference
Cancer type	Animal model	IC_50_/Working dose	Mechanisms	Primary outcomes	
Pancreatic	nu/nu male nude mice	5 mg/kg body weight of mice	↑Bax ↓Bcl‐2	↑Apoptosis ↓Tumor formation	Sun et al. (2014)
	Athymic (nu/nu) male nude mice	6 μg/g body weight of mice	—	↓Tumor volume ↓Migration	Lv et al. (2014)
Breast	Female BALB/c mice	5 mg/kg	↑Bax, ↓Bcl‐2	↑Apoptosis	Mohsenikia et al. (2013)
Prostate	Male nude mice	5 mg/kg	↓Cell cycle transition, ↓level of cyclin D1/E1, CDK2, CDK4, CDK6, ↓p21	↓Tumor growth ↑Apoptosis	Pan et al. (2016)
Colorectal	Male BALB/c nude mice	5 mg/kg	↑Bax, ↓Bcl‐2, ↓Ki‐67	↓Tumor growth ↑Tumor weight ↑Apoptosis	Yan et al. (2020)
Choriocarcinoma	Athymic nude mice	—	↑Bax, ↓Bcl‐2, and PCNA in xenograft mice	↓Tumor growth ↓Tumor weight ↑Apoptosis	Gu et al. (2021)

Abbreviations: ↑, increase; ↓, decrease; 4E‐BP1, Eukaryotic translation initiation factor 4E‐binding protein 1; AML‐193, Acute Myeloid Leukemia cell line 193; ASK1, Apoptosis signal‐regulating kinase 1; Bax, Bcl‐2‐associated X protein; Bcl‐2, B‐cell lymphoma 2; CD44, Cell surface glycoprotein CD44; CDK2, Cyclin‐Dependent Kinase 2; CDK4, Cyclin‐Dependent Kinase 4; CDK6, Cyclin‐Dependent Kinase 6; Cyclin D1/E1, Cell cycle proteins Cyclin D1 and Cyclin E1; DU145, prostate cancer cell line; EC9706, Esophageal cancer cell line; Eca109, Esophageal carcinoma cell line 109; E‐cadherin, epithelial cadherin; EMMPRIN, Extracellular matrix metalloproteinase inducer; eNOS, Endothelial Nitric Oxide Synthase; ERK, Extracellular signal‐regulated kinase; GAS5, Growth arrest‐specific 5 (non‐coding RNA); GPI, glucose‐6‐phosphate isomerase; HCT‐116, colorectal cancer cell line; HDAC1, Histone deacetylase 1; Hep G2, Liver hepatocellular cells; HIF‐1α, hypoxia‐inducible factor 1‐alpha; IC50, half maximal inhibitory concentration; JAK/STAT, Janus kinase/signal transducers and activators of transcription; JEG‐3, Choriocarcinoma cell line; LAMP‐2, Lysosomal‐associated membrane protein 2; LC3‐II, Microtubule‐associated protein 1A/1B‐light chain 3; LDHA, Lactate dehydrogenase A; MIA PaCa‐2, Pancreatic carcinoma cell line 2; miR‐138, MicroRNA‐138; miR‐16, MicroRNA‐16; miR‐18a, MicroRNA‐18a; miR‐21, MicroRNA‐21; MMP‐2, 9, matrix metalloproteinase‐2 and ‐9; mTOR, mechanistic target of rapamycin; NF‐κB, Nuclear factor kappa‐light‐chain‐enhancer of activated B cells; NSCLC, non‐small cell lung cancer; PANC‐1, Pancreatic cancer cell line 1; PATU‐8988, Pancreatic tumor cell line 8988; PC‐3, prostate cancer cell line; PCNA, proliferating cell nuclear antigen; PKLR, Pyruvate kinase L/R; RKO, colorectal adenocarcinoma cell line; ROS, Reactive oxygen species; STAT3, Signal transducer and activator of transcription 3; SW1990, Pancreatic cancer cell line; SW480, SW620, Colorectal adenocarcinoma cell lines; TBP‐2, Thioredoxin‐binding protein 2 (also known as TXNIP), thioredoxin‐interacting protein; TPI1, Triosephosphate isomerase 1; VEGF, Vascular endothelial growth factor; Vimentin, Type III intermediate filament protein; Wnt/β‐catenin, Wnt signaling pathway involving β‐catenin.

### 6.1 Anticancer mechanisms of α‐solanine: Apoptosis, autophagy, angiogenesis, and metastasis inhibition

α‐Solanine's effectiveness against cancer has been attributed to its ability to trigger multiple cellular mechanisms, and some pharmacological studies have examined these mechanisms, focusing on the intrinsic pathway of apoptosis, the interplay between autophagy and apoptosis, the suppression of angiogenesis and metastasis, and the modulation of key signaling pathways.

#### 6.1.1 Apoptosis induction

Apoptosis, a programmed cell death mechanism in multicellular organisms, involves a series of biochemical events that lead to distinct cellular changes such as shrinkage, nuclear fragmentation, and ultimately cell death; and this process is vital for development and maintaining bodily health by removing old, unnecessary, or damaged cells (Vitale et al., 2023). Research shows that α‐solanine predominantly activates the mitochondria‐mediated intrinsic pathway of apoptosis in cancer cells. This pathway is marked by the downregulation of the anti‐apoptotic protein Bcl‐2 and the translocation of the pro‐apoptotic protein Bax to the mitochondrial membrane. This translocation results in the permeabilization of the mitochondrial outer membrane (MOMP), leading to the release of cytochrome C into the cytoplasm. This release initiates caspase activation, culminating in cell death. The balance between Bax and Bcl‐2 is crucial in regulating mitochondrial membrane permeability transition pore (MPTP) formation and determining the cell's response to death signals (Nandi et al., 2019).

#### 6.1.2 α‐Solanine in autophagy and drug resistance

Autophagy is a cellular process where cells degrade and recycle their own components, helping to maintain cellular health by removing damaged proteins and organelles. Apart from apoptosis, autophagy is also regarded as a therapeutic target for treating drug‐resistant cancer cells (Bhuia et al., 2023; Yun et al., 2020). The complicated association between apoptosis and autophagy is currently being investigated as a therapeutic target in cancer. It has been noted that autophagy switches to apoptosis, and inhibition of autophagy delays apoptosis (Nandi et al., 2022). Hence, exploring the relationship between apoptosis and autophagy is emerging as a constructive strategy toward the development of highly selective and competent anticancer drugs. Most studies have demonstrated the activation of apoptosis as the underlying mechanism for the anticancer activity of α‐solanine. One of the studies reported α‐solanine as a potent inducer of autophagy, which might act in parallel or synergistically with apoptosis to impart its cytotoxic effect.

#### 6.1.3 Impact on angiogenesis

Angiogenesis, the formation of new blood vessels, is critical for tumor growth and metastasis; under a hypoxic environment, the elevated expression level of prime regulators of the angiogenesis process, such as activator of transcription 3 (phosphorylated‐STAT3), hypoxia‐inducible factor‐1 and 1α, nuclear transcription factor, and the phosphorylated signal transducer, was noted (Liu et al., 2023). Inhibition of VEGF, a proangiogenic growth factor, suppresses the angiogenesis of tumors and induces the formation of new blood vessels, which is very critical to invasiveness, metastasis formation, and/or tumor growth (Liu et al., 2023; Tu et al., 2023). α‐Solanine decreased the amount of VEGF and E‐cadherin and also suppressed the activation of STAT3, phosphor‐ERK1/2, and HIF‐1α signaling pathways in pancreatic cancer cells. Together, the results provided novel evidence that α‐solanine has a strong anti‐metastatic and anticancer effect via the ERK1/2‐HIF‐1α and STAT3 signaling pathways (Wen et al., 2016).

#### 6.1.4 Impact on metastasis

Metastasis is the process by which cancer cells spread from the original tumor site to other parts of the body, forming new tumors in distant organs, and is a hallmark of advanced‐stage cancers (Gerstberger et al., 2023). The phosphatidylinositide‐3 kinase (PI3K)/Akt signaling pathway is tightly engaged in the regulation of cellular metastasis; hence, the invasion, metastasis, and expansion of tumor cells can be restricted by dysregulating the PI3K/Akt and MAPK signaling pathways (Kciuk et al., 2022). α‐Solanine has been reported to restrict the phosphorylation of PI3K, Akt, and ERK1/2. The results depicted that the inhibitory effect of α‐solanine on gene expression involved in epithelial–mesenchymal transition and proteolytic activation was solely due to its efficacy to prevent ERK1/2 and PI3K/Akt signaling pathways (Shen et al., 2014). Hence, the results altogether suggest that α‐solanine is a potential inhibitor of the invasion of cancer cells.

Table 3 and Figure 4 outline the primary anticancer mechanisms and signaling pathways influenced by α‐solanine, based on recent studies.

**TABLE 3:** Summary of anticancer mechanisms of α‐Solanine.

Mechanism	Description	Effect	Signaling pathways involved	Ref
Apoptosis Induction	Activates the mitochondria‐mediated intrinsic pathway of apoptosis in cancer cells, a crucial mechanism for inducing programmed cell death	↓Bcl‐2, ↑Bax ↓MOMP, ↑discharge of Cytochrome C ↑caspase activation and cell death	Bax/Bcl‐2 ratio regulates ↓MPTP formation	Nandi et al. (2019)
Autophagy and Drug Resistance	Regarded as a therapeutic target for drug‐resistant cancer cells, with a complex relationship between apoptosis and autophagy	↑Autophagy Potentially acting in parallel or synergistically with apoptosis for cytotoxic effects	—	Yun et al. (2020), Bhuia et al. (2023), Nandi et al. (2022)
Angiogenesis Inhibition	Critical in tumor growth and metastasis; α‐Solanine inhibits angiogenesis under hypoxic conditions	↓VEGF, ↓E‐cadherin ↓STAT3, ↓ phosphor‐ERK1/2, ↓HIF‐1α s	↓ERK1/2‐HIF‐1 ↓STAT3	Wen et al. (2016)
Metastasis Inhibition	α‐Solanine impacts the PI3K/Akt signaling pathway, which is crucial in cellular metastasis	↓PI3K, ↓Akt, ↓ERK1/2 ↓Genes involved in epithelial–mesenchymal transition and proteolytic activation	↓PI3K/Akt ↓MAPK	Kciuk et al. (2022), Shen et al. (2014)

Symbols and abbreviations: ↑, increase; ↓, decrease; Akt, Protein Kinase B; EMT, Epithelial–Mesenchymal Transition; ERK1/2, Extracellular Signal‐Regulated Kinases 1 and 2; HIF‐1α, Hypoxia‐Inducible Factor 1‐alpha; MAPK, Mitogen‐Activated Protein Kinases; MOMP, Mitochondrial Outer Membrane Permeabilization; MPTP, Mitochondrial Membrane Permeability Transition Pore; PI3K, Phosphoinositide 3‐Kinases; STAT3, Signal Transducer and Activator of Transcription 3; VEGF, Vascular Endothelial Growth Factor.

### 6.2 In vitro studies

#### 6.2.1 Non‐small cell lung cancer (NSCLC)

Non‐small cell lung cancer, encompassing subtypes like adenocarcinoma, squamous cell carcinoma, and large cell carcinoma, is a prevalent form of lung cancer noted for its slower growth and spread compared to small cell lung cancer. This particular nature of NSCLC is crucial in the context of pulmonary complications, especially parapneumonic pleurisy. Parapneumonic pleurisy, commonly linked with severe lung infections (GBD 2019 Antimicrobial Resistance Collaborators, 2022), tends to present differently in patients with NSCLC, significantly affecting their clinical management and treatment pathways (Alduais et al., 2023; Calina et al., 2016; Ungureanu et al., 2017). α‐Solanine‐treated A549 cells exhibited an increased accumulation of LC3‐specific puncta as well as elevated expression of LC3‐II at a minimum concentration of 10 μM. Other key regulatory proteins of autophagy were also examined, and the results clearly showed that treatment with α‐solanine enhanced the turnover of Beclin, Atg5, and lysosome‐associated membrane protein 2 (LAMP‐2) in a time‐dependent manner. Considering the Akt/mTOR pathway is known to be involved in the regulation of autophagy and apoptosis, *α‐*solanine was found to block the pathway by downregulating the phosphorylation of Akt, mammalian target of rapamycin (mTOR), and eukaryotic translation initiation factor 4E‐binding protein 1 (4E‐BP1), leading to autophagy. Studies also demonstrated that in regulating autophagy, α‐solanine can induce oxidative damage and increase the expression of endoplasmic reticulum (ER) stress proteins (X‐box‐binding protein 1, BiP, PERK, activating transcription factor 6 (ATF6), ATF4, inositol‐requiring transmembrane kinase/endonuclease 1, and CCAAT‐enhancer‐binding protein (C/EBP)‐homologous protein). This suggests that α‐solanine can induce autophagy to exert anti‐proliferative activity by triggering ER stress and blocking the Akt/mTOR signaling pathway. Furthermore, increased cleavage of caspase 3 and PARP was also noted in A549 cells, pointing toward apoptosis induction (Hasanain et al., 2015). The effect of α‐solanine was examined on non‐small cell lung cancer (NSCLC) and showed that α‐solanine increased the apoptotic rate and retarded the cell viability of both A549 and PC‐9 cells in a dose‐dependent as well as time‐dependent manner. Further studies have demonstrated that α‐solanine significantly suppresses the migration and invasion of A549 and PC‐9 cells. The study also showed that α‐solanine might inhibit the survival and growth of NSCLC by regulating glycolysis‐related pathways, as the expression of proteins such as GPI (glucose phosphate isomerise), ALDOA (aldolase fructose‐bisphosphate A), TPI1 (triosephosphate isomerase 1), PKLR (Pyruvate Kinase L/R), LDHA (lactate dehydrogenase deficiency), and ALDH3 (human aldehyde dehydrogenase 3) were found to be downregulated (Zou et al., 2022).

#### 6.2.2 Digestive cancers

##### Esophageal cancer

Esophageal cancer arises in the esophagus and typically manifests as squamous cell carcinoma or adenocarcinoma. Commonly presenting with difficulty swallowing and weight loss, its treatment varies from surgery to chemotherapy and radiation based on its progression (Sheikh et al., 2023). The anticancer efficacy of α‐solanine was also tested against human esophageal carcinoma cells, and the results revealed that it elevated the apoptotic rate and inhibited cell growth as well as proliferation in a dose‐dependent manner in human esophageal EC9706 and Eca109 cancer cells. Additionally, it also suppressed the cellular migration and invasion of esophageal cancer cells by downregulating the expression levels of tumor metastasis‐related proteins, MMP‐2 and MMP‐9, and conversely increasing the expression levels of E‐cadherin (Wang, Sun, et al., 2016).

##### Hepatic cancer

Liver cancer, primarily hepatocellular carcinoma, is a malignancy originating in the liver; major risk factors include chronic hepatitis B or C infection, fatty liver disease, cirrhosis, and lifestyle factors like excessive alcohol consumption (Lazarus et al., 2023). Tobacco smoking has also been identified as a significant risk factor, contributing to the disease's development through various carcinogenic mechanisms (Jain et al., 2021). It was reported that Hep G2 cells treated with α‐solanine exhibited disruption of mitochondrial membrane potential, thereby opening MPTP and increasing the concentration of Ca^2+^ in the cytoplasm of tumor cells to execute apoptosis (Gao et al., 2006). Solanine elevated the rate of apoptosis in Hep G2 cells to a maximum level of 32.2%. It also inhibited the proliferation of Hep G2 cells by arresting the cell cycle at the S phase with the concomitant absence of cell population in the G2/M phase and induced downregulation of the anti‐apoptotic protein Bcl‐2 (Ji et al., 2008). In vitro administration of α‐solanine in Hep G2 cells has successfully induced their apoptosis. The cellular apoptotic rate was remarkably increased in the α‐solanine‐treated Hep G2 cells as compared with the control cells. The study demonstrated that the mode of action behind the solanine‐induced apoptosis in Hep G2 cells was increased generation of reactive oxygen species (ROS), mainly OH^·^ and H_2_O_2_, in a mitochondrial‐dependent and independent manner. α‐Solanine also elevated the expression and kinase activity of apoptosis signal‐regulating kinase 1 (ASK1) and thioredoxin binding protein 2 (TBP‐2), which in turn activated the JNK and p38 signaling pathways. It further inhibited the level of proliferation‐associated proteins, like histone deacetylase 1 (HDAC1), thus contributing to the apoptosis of Hep G2 cells (Meng et al., 2016).

Lin et al. (2020) explored that α‐solanine remarkably attenuated the proliferation of Hep G2 induced by the acetylcholine signal. It reduced the migratory and metastasis activity of liver cancer cells caused by acetylcholine induction. Solanine treatment abolished the promotion of vimentin, E‐cadherin, MMP‐2, and 9 in Hep G2 cells induced by acetylcholine, suggesting that solanine has effectively suppressed metastasis, attenuating epithelial–mesenchymal transition (EMT) (Lin et al., 2020).

##### Pancreatic cancer

Pancreatic cancer, a highly aggressive malignancy with a poor prognosis, is characterized by rapid progression and resistance to conventional therapies, often presenting late with non‐specific symptoms that complicate early diagnosis and effective treatment (Jiang et al., 2023). SW1990 and Panc‐1 cells treated with solanine increased the expression level of p53 and Bax, coupled with the downregulation of Bcl‐2 levels, thereby opening the MPTP. Furthermore, an increased level of cytochrome c and Smac was also noted in the cytosol, followed by the cleavage of caspase‐3 zymogen into an activated form. Hence, solanine inhibited pancreatic cancer cell growth through caspase‐3‐dependent mitochondrial apoptosis. Solanine also suppressed pancreatic cancer metastasis by decreasing the expression of MMP‐2 and MMP‐9 (Sun et al., 2014).

Another study reported that α‐solanine inhibited the cellular proliferation, migration, and invasion of PANC‐1, SW1990, and MIA PaCa‐2 cells in a dose‐dependent manner. Further studies were also conducted in PANC‐1 cells to observe the expression of metastasis‐associated molecules, and the data precisely exhibited the downregulation of CD44, MMP‐2/9, eNOS, an extracellular inducer of matrix metalloproteinase (EMMPRIN), and E‐cadherin. The investigated mode of action confirmed the role of suppressed Akt/mTOR, Wnt/β‐catenin, and JAK/STAT pathways in the cellular invasion. Moreover, the results also revealed that α‐solanine potentially inhibited angiogenesis by restraining the expression level of VEGF and contemplating the tube formation of endothelial cells (Lv et al., 2014).

##### Colorectal cancer

Colorectal cancer is a type of cancer that begins in the colon or rectum, part of the large intestine, and the digestive system; it is characterized by the uncontrolled growth of cells in the colon or rectal lining (Irfan et al., 2022; Zlatian et al., 2015). α‐Solanine showed cytotoxic effects on SW480, SW620, and HT‐29. It also inhibited their proliferation, migration, and invasion and promoted apoptosis by arresting the cell cycle (Ni et al., 2018). S100P, which is involved in the regulation of various cellular processes such as cell cycle differentiation and progression, has been downregulated by α‐solanine in colorectal cancer (Ni et al., 2018). α‐Solanine showed potential anticancer activity against colorectal cancer cell lines, where it inhibited the growth of RKO cells in a dose‐ and time‐dependent manner. Moreover, α‐solanine induced apoptosis in RKO cells by causing cell cycle arrest at the G_0_/G_1_ phase coupled with downregulated expression of CCND1 and CDK 4‐induced ROS generation, which contributed to caspase activation of caspase‐3, ‐8, and ‐9. Additionally, at 20.84 μM, α‐solanine inhibited the invasion and adhesion of RKO cells by suppressing the activities of MMP‐2 and MMP‐9. α‐Solanine has also inhibited the proliferation of HCT‐116 cells in a dose‐dependent manner and induced apoptosis by activating caspases (caspase‐3, ‐8, and ‐9). In addition, inhibition of the migration and invasion of HCT‐116 cells was also observed upon treatment with α‐solanine (Yan et al., 2020).

#### 6.2.3 Prostate cancer

Prostate cancer, one of the most common types of cancer among men, often grows slowly and is initially confined to the prostate gland, where it may not cause serious harm, but in some cases, it can grow more rapidly and spread to other parts of the body, necessitating a range of treatments, from localized therapies to more aggressive interventions (Lowrance et al., 2023).

A further study investigated the inhibitory mechanism of solanine in vitro on cancer development in a cultured human prostate cancer cell line, DU145. The results demonstrated that α‐solanine remarkably inhibited cellular growth by modulating the protein levels of major cell cycle proteins, like Cyclin D1, Cyclin E1, CDK2, CDK4, CDK6, and p21. α‐Solanine is further noted to induce apoptosis in DU145 cells by increasing the production of ROS and the phosphorylated level of p38, thereby activating the p38 MAP kinase pathway (Pan et al., 2016).

α‐Solanine is also used as an adjuvant therapy for suppressing the invasion of prostate cancer cells. The results showed that α‐solanine not only reduced the viability of PC‐3 cells but also the invasion of cancer cells when treated with non‐toxic doses. α‐Solanine has potentially reduced the expression of mesenchymal marker vimentin while elevating the negative regulators of MMPs, namely tissue inhibitors of metalloproteinase (TIMP‐1 and 2), reversion‐inducing cysteine‐rich proteins with kajal motifs (RECK), and epithelial marker E‐cadherin, suggesting the suppression of epithelial–mesenchymal transition (EMT). It also downregulated other migration‐associated molecules like EMMPRIN, MMP‐2, and MMP‐9 (Shen et al., 2014).

#### 6.2.4 Skin melanoma

In a recent study, α‐solanine has productively suppressed the invasion and migration of A2058 melanoma cells in a dose‐dependent manner at non‐toxic concentrations (9.2, 13.8, and 18.4 μM). This attributes top inhibition of the effects of matrix metalloproteinases (MMP‐2 and 9) that are associated with the migration and invasion of the melanoma cell line. Other biochemical assays also confirmed the anti‐metastatic activity of α‐solanine by reducing the activity of the nuclear level of nuclear factor kappa B (NF‐κB) and phosphorylation of PI3K, Akt, and JNK (Lu et al., 2010).

#### 6.2.5 Acute myeloid leukemia

Studies on AML‐193 acute myeloid leukemia (AML) cells demonstrated that α‐solanine caused dose‐dependent pro‐apoptotic effects and morphological alterations in neoplastic cells. It also altered the expression of pro‐apoptotic genes encoding Bax. α‐Solanine exerts its anti‐proliferative effect by targeting the miR‐16/Bcl‐2 axis, where miR‐16 is upregulated and Bcl‐2 is downregulated (Zheng et al., 2020). Moreover, α‐solanine inhibited cell viability and accelerated the apoptotic pathway in human choriocarcinoma cells. It also reduced the invasion and migration of JEG‐3 cells by degrading the active forms MMP‐2 and 9 (Gu et al., 2021).

### 6.3 In vivo studies

#### 6.3.1 Pancreatic cancer

The influence of solanine in the in vivo model was noted in the study using nu/nu nude mice injected with 4 × 10^6^ SW1990 cells. When treated with solanine at a dose of 5 mg/kg, a reduction in tumor size coupled with modulation of the mitochondrial‐mediated signaling pathway, including the ratio of Bax and Bcl‐2, was noted (Sun et al., 2014). Another group of researchers also administered α‐solanine (6 μg/g for 2 weeks) in the xenograft mice model, which is subcutaneously injected with 5 × 10^6^ PANC‐1 cells. The treatment decreased tumor volume as well as weight by 61% and 43%, respectively, along with downregulating the expression of MMP‐2/9, PCNA, and VEGF (Lv et al., 2014).

#### 6.3.2 Colorectal cancer

Colorectal cancer, which originates in the colon or rectum, is influenced by genetic actors; long non‐coding RNAs play a significant role in this cancer by enabling cells to evade apoptosis, thereby contributing to tumor progression and treatment resistance (Garzoli et al., 2022; Irfan et al., 2022). α‐solanine inhibited tumor growth in BALB/c nude mice, as demonstrated by tumor volume and tumor weight. α‐Solanine also inhibited the expression of Ki‐67, a proliferation marker, as detected by immunohistochemistry, indicating that α‐solanine can inhibit cell proliferation in vivo (Yan et al., 2020). When 2 × 10^6^ RKO human colorectal cancer cells were introduced in BALB/c nude mice and a 5 mg/kg dose of α‐solanine was applied every day for 12 days, a dramatic reduction in the size of the tumor, tumor volumes, and weights of treated mice relative to control animals were observed. The major reason for tumor cell proliferation inhibition in α‐solanine‐treated animals was the suppression of the proliferative marker Ki‐67, which accelerated apoptosis. Apoptosis was induced in xenograft tumors of α‐solanine‐treated mice by downregulating the expression of Bcl‐2 and proliferative cell nuclear antigen (PCNA) while activating the expression of Bax (Gu et al., 2021).

#### 6.3.3 Prostate cancer

Prostate cancer is a disease where cancer cells form in the prostate gland in men; it ranges from slow‐growing to more aggressive forms that can spread quickly; early detection often leads to better treatment outcomes (Rebello et al., 2021). Administration of 5 mg/kg solanine for a month in xenograft athymic nude mice induced with human prostate cancer cells DU145 significantly reduced tumor cell proliferation and prevented cell cycle transition from the G_1_ phase to the S phase. The fundamental mechanism was associated with the modulation of the primary cell cycle proteins that are accountable for the seamless advancement of the cell cycle. This includes the downregulation of the cyclin D1/E1 complex, followed by suppression of CDK 2/4/6 and upregulation of the p21 protein (Pan et al., 2016).

#### 6.3.4 Breast cancer

Breast cancer is a malignancy that forms in breast tissues, predominantly affecting women, and early detection is crucial for effective treatment (Storme, 2023). α‐Solanine effectively targeted the mouse mammary carcinoma cells and reduced their growth significantly. It was observed that intravenous administration of a 5 mg/kg dose of α‐solanine to female BALB/c mice suppressed tumor growth by controlling the ratio of Bax and Bcl‐2, leading to the induction of apoptosis in tumor cells. Animals treated with α‐solanine also showed a lower level of proliferation and angiogenic markers (Mohsenikia et al., 2013).

## 7 ANTICANCER EFFICACY OF COMBINATIONS OF Α‐SOLANINE WITH CONVENTIONAL DRUGS TO ELEVATE TOLERANCE TO CHEMOSENSITIVITY AND RADIOSENSITIVITY

Hostile effects of conventional cancer therapies are the most significant concern encountered by cancer patients during their ailment and notably compromise their standard of living (Baldo et al., 2018). In the process of cancer treatment, resistance to drugs is an intractable issue and a prime hurdle for patients experiencing extended chemotherapy. Clinical resistivity against anticancer agents occurs during the time of drug initiation, as well as during the treatment procedure and following relapse (Quesada et al., 1996). Cancers susceptible to drugs generate resistance against chemotherapy treatment through several mechanisms described, such as usage of alternate metabolic pathways, involvement of altered molecules in apoptosis initiation, inadequate invigoration of the drug, DNA damage repair and suppressed drug accumulation. Due to several negative impacts of chemotherapy, an elevated dose causes rigorous side effects (Gottesman, 2002). Along with chemosensitivity, radiosensitivity is another important modality used in treating a variety of cancers, but clinically non‐satisfactory because of only a 10–30% survival rate for 5 years and the high recurrence rate of tumors (Baskar et al., 2012). Consequently, utilizing a combination of chemotherapeutic drugs with several mechanisms could synergistically heighten therapeutic competency and be widely utilized for a variety of cancer types (Glasgow & Chougule, 2015). Essentially, the choice of taking multiple drugs seems to lower the doses, which might lead to low resistance and the maintenance of either higher or the same efficacy (He et al., 2015; Liboiron & Mayer, 2014).

This study's findings indicate that α‐solanine may improve patient resilience to chemotherapy and radiotherapy while diminishing their harmful side effects. Various in vitro studies have shown that α‐solanine treatment in esophageal cancer cells lines like EC9706 and KYSE30, followed by irradiation, increased the effectiveness of radiation therapy in inhibiting these cancer cells. Further investigations involving microRNAs revealed that α‐solanine elevated the expression of miR‐138, a known tumor suppressor, and concurrently reduced the levels of survivin, a key inhibitor of apoptosis and a major target of miR‐138, in these cell lines. Consequently, α‐solanine enhanced the radiosensitivity of cancer cells by increasing miR‐138 accumulation. This suggests that the combination of α‐solanine with the miR‐138/Survivin axis might be a potent radiosensitizer for esophageal cancer cells (Wang, Wu, et al., 2016). Comparable effects were observed in lung adenocarcinoma cells. In cell lines such as H1299 and A549, α‐solanine not only inhibited cell migration and invasion but also increased their sensitivity to chemotherapy and radiotherapy. This study also highlighted that α‐solanine significantly altered miR‐138 expression and downregulated focal adhesion kinase (FAK), a direct target of miR‐138, in A549 and H1299 cells. These findings suggest that α‐solanine targets lung adenocarcinoma through its impact on miR‐138 and FAK expression (Zhang et al., 2016). Overall, α‐solanine has shown considerable anti‐cancer properties and has been effective in enhancing chemotherapeutic and radiotherapeutic responsiveness across various human cancers.

A study explored α‐solanine's effect on enhancing radiosensitivity in prostate cancer. In vitro experiments showed that α‐solanine reduced cell viability and triggered apoptosis in human prostate cancer cells. Notably, α‐solanine counteracted the effects of ectopically expressed growth arrest‐specific 5 (GAS5), which typically inhibits cell viability and promotes apoptosis, by overexpressing miR‐18a in DU145 and PC‐3 cells. During treatment, GAS5 was observed to regulate miR‐18a expression via target binding. Therefore, α‐solanine was effective in inhibiting cell proliferation and boosting radiosensitivity through the upregulation of the GAS5/miR‐18a pathway in these cell lines (Yang et al., 2019). Another study highlighted α‐solanine's role in increasing the sensitivity of leukemia cells to Adriamycin, an anthracycline widely used in cancer treatment (Yi, Jia, Wang, et al., 2018; Yi, Jia, Zhu, et al., 2018). α‐Solanine enhanced the susceptibility and reduced the resistance of leukemia cells to Adriamycin by downregulating the expression of the multidrug resistance‐associated protein 1 (MRP1), a member of the ABC transporter protein family known for reducing intracellular drug accumulation, thereby diminishing the efficacy of chemotherapeutic agents (Yi, Jia, Wang, et al., 2018). Furthermore, the collaborative impact of combining the natural alkaloid α‐solanine with a low dose of cisplatin was examined in human hepatocellular carcinoma cells. This combination synergistically increased the apoptotic effect of cisplatin on HepG2 cells, inducing DNA fragmentation, cell cycle arrest, and activating regulators of both intrinsic and extrinsic apoptosis pathways. This induced apoptosis was also mediated by altering miR‐21 expression (El‐Daly et al., 2019).

Table 4 provides a comprehensive overview of the enhanced efficacy observed in various cancer types when α‐solanine is combined with chemotherapy and radiotherapy, highlighting key findings in cell line models, the mechanisms involved, and the outcomes of these combined treatments as documented in several studies.

**TABLE 4:** Synergistic effects of α‐solanine in combination with standard cancer therapies: enhanced chemosensitivity and radiosensitivity across various cancer models.

Cancer type	Combined treatment	Cell lines/models	Effects	Mechanisms	Reference
Esophageal cancer	α‐Solanine + Radiotherapy	EC9706, KYSE30	↑Radiosensitivity irradiation inhibition	↑miR‐138 ↓survivin	Wang, Wu, et al. (2016)
Prostate cancer		DU145, PC‐3	↓Cell viability ↑Apoptosis ↑Radiosensitivity	↑GAS5/miR‐18a pathway	Yang et al. (2019)
Lung adenocarcinoma	α‐Solanine + Chemo/Radiotherapy	H1299, A549	↓Migration ↓Invasion ↑Chemosensitivity ↑Radiosensitivity	↓miR‐138 ↓FAK	Zhang et al. (2016)
Leukemia	α‐Solanine + Adriamycin	K562/ADM	↑Sensitivity, ↓resistance to Adriamycin	↓MRP1 expression	Yi, Jia, Zhu, et al. (2018)
Hepatocellular carcinoma	α‐Solanine + Cisplatin	HepG2	↑Apoptotic effect ↑DNA fragmentation ↑Cell cycle arrest	↑miR‐21 expression ↑apoptosis	El‐Daly et al. (2019)

Symbols and abbreviations: ↑, increase; ↓, decrease; FAK, Focal Adhesion Kinase; GAS5, Growth Arrest‐Specific 5; miR‐138, miR‐18a, miR‐21, Specific microRNAs (miRNAs) involved in the studies; MRP1, Multi‐Drug Resistance‐Associated Protein 1.

## 8 TOXICITY, SAFETY DATA AND SIDE EFFECTS

α‐Solanine is recognized as a broadly toxic agent against various entities, including viruses, bacteria, fungi, animals, insects, and humans, functioning as a defensive compound in plants (Wang et al., 2022). Symptoms associated with its ingestion, even in minimal quantities, typically manifest within 8–12 h (Mohsenikia et al., 2016). Its toxicity in animal models varies based on dosage, species, and administration method (Barceloux, 2009). Symptoms of solanine poisoning often include gastrointestinal and neurological disturbances, such as vomiting, headaches, and flushing (Phillips et al., 1996). A particular study assessed the impact and pharmacokinetic properties of potato glycoalkaloids (α‐chaconine and α‐solanine) in humans following oral intake. It was observed that single doses up to 90.2 mg of total glycoalkaloid (TGA) did not induce acute systemic effects. Nonetheless, a dose of 1.25 mg TGA/kg body weight caused minor gastrointestinal issues in one of the 14 adult participants, likely due to glycoalkaloid toxicity. The study also revealed that potato glycoalkaloids could accumulate in the human body, as their elimination typically exceeds 1 day (Mensinga et al., 2005). Solanine is metabolized in the stomach to a less harmful form, solanidine, and is primarily excreted through feces and, to a lesser extent, in urine (Norred et al., 1976). Further research indicates that solanidine regularly appears in the bloodstream of human volunteers, suggesting that α‐solanine has a relatively extended serum half‐life (Chain et al., 2020).

Bergers and Alink (1980) conducted experiments to investigate the toxicological effects of glycoalkaloids, specifically α‐solanine and tomatine, using cardiac cell cultures from rats aged 1–2 days. Their findings indicated that cellular beating ceased within minutes when the cultures were exposed to 80 μg/mL of α‐solanine and 40 μg/mL of tomatine. At reduced concentrations of 40 μg/mL for α‐solanine and 20 μg/mL for tomatine, there was a significant increase in contraction frequency for at least 2 h. High doses of α‐solanine (100 mg/kg and above) were lethal in animal studies. Moreover, administering α‐solanine at 20 mg/kg or more for a week led to changes in fluid regulation, elevation of RBC, Hct, and Hgb levels, along with weight loss and ascites formation. Dehydration was also observed in mice treated with these glycoalkaloids. Furthermore, high doses of solanine notably decreased liver mass and alkaline phosphatase levels, indicating potential kidney and liver dysfunction in vivo (Mohsenikia et al., 2013). Animals exposed to lower quantities of α‐solanine showed comparatively fewer adverse effects than those exposed to higher doses.

## 9 LIMITATIONS OF α‐SOLANINE AS POTENTIAL THERAPEUTIC AGENT IN ONCOLOGY

The application of α‐solanine in oncology, though promising due to its potential anti‐cancer properties is faced with several limitations:

- One of the primary limitations is its narrow therapeutic index; the margin between therapeutic and toxic doses of α‐solanine is small, making it challenging to administer safely without risking toxicity.
- There is a significant lack of comprehensive clinical studies/trials to establish the safety and efficacy of α‐solanine in cancer treatment; most of the existing studies are preclinical, conducted in vitro or in animal models, which may not accurately translate to human therapy.
- α‐Solanine can cause a range of toxic effects, including gastrointestinal distress, neurological symptoms, and, in severe cases, respiratory failure (Langkilde et al., 2008). This toxicity limits its clinical use, as the risks may outweigh the potential therapeutic benefits for cancer patients.
- The bioavailability of α‐solanine, especially when administered orally, is variable and can be influenced by factors such as food interactions and individual patient metabolism (Abuhelwa et al., 2017); this variability complicates the establishment of a consistent and effective dosing regimen.
- The mechanisms by which α‐solanine exerts its anti‐cancer effects are not fully understood; without a clear understanding of these mechanisms, it is difficult to predict its efficacy and safety in different types of cancer and to develop targeted treatment strategies.
- Cancer patients often receive multiple medications (GBD 2019 Colorectal Cancer Collaborators, 2022), and the potential interactions of α‐solanine with other drugs used in oncology are not well studied; these interactions could affect the efficacy of treatment or increase the risk of adverse effects.
- The specificity and selectivity of α‐solanine for cancer cells over normal cells are not well established; without high selectivity, α‐solanine could harm healthy cells, leading to additional complications in cancer patients.
- The pharmacokinetics of α‐solanine, including its metabolism and excretion, present challenges in oncology settings. Patients with cancer, particularly those with liver or kidney impairments, may have altered pharmacokinetics, affecting the drug's behavior and necessitating careful dose adjustment.

In summary, while α‐solanine holds potential in oncology, its clinical application is significantly limited by its narrow therapeutic index, toxicity concerns, lack of clinical trials, variable bioavailability, and insufficient understanding of its mechanisms. Further research is necessary to overcome these limitations and safely integrate α‐solanine into cancer treatment protocols.

## 10 CONCLUSION AND FUTURE PERSPECTIVES

The review highlights the anti‐carcinogenic efficacy of α‐solanine. It summarizes the most recent in vitro and in vivo studies on various types of human cancers with this compound, its anti‐cancer potency, and the mode of action. This steroidal alkaloid is a budding therapeutic for treating different types of carcinogenic tumors. The spectrum of its cytotoxicity is considered broad and diverse against a wide variety of cancer cell lines. α‐Solanine promotes apoptosis by activating or blocking several cell signaling pathways. It triggers multiple cell mechanisms, such as microtubule interactions or probable transformations in Bax and Bcl‐2 expression, which can lead to the stoppage of the progression of cancer and its further development. α‐Solanine also significantly suppresses angiogenesis by controlling multiple angiogenic factors. Another application of these compounds is combinational therapy, in which they are applied with other existing potential drugs and therapies for the improvement of their efficiency. This may enhance the quality of treatment for patients who are suffering from severe cancer by reducing resistance to the existing drugs. In this article, the analysis of all available data on α‐solanine provides a deeper understanding of the compound as a potential anticancer pharmaceutical/therapeutic agent. Although a vast number of toxicological and pharmacological research have established α‐solanine as a potential anti‐tumor agent, there is still a lack of large‐scale clinical studies, and many more explorations are needed to understand the mechanisms regarding the biological implications and also to estimate the toxicological effects associated with carcinogenic and normal cells, which progress toward cancer prevention. However, research regarding the development and study of solanine and its derivative preparations includes huge economic potential, together with the development of new scientific doorways. Because of the outlined therapeutic potential, further studies on the structural modification of α‐solanine to maximize potency and reduce potential toxicity in humans are well merited.

## AUTHOR CONTRIBUTIONS

**Sudeshna Nandi:** Data curation (equal); investigation (equal); methodology (equal); writing – original draft (equal); writing – review and editing (equal). **Rimpa Sikder:** Data curation (equal); methodology (equal); writing – original draft (equal); writing – review and editing (equal). **Anish Nag:** Data curation (equal); investigation (equal); methodology (equal); writing – original draft (equal); writing – review and editing (equal). **Somanjana Khatua:** Data curation (equal); investigation (equal); methodology (equal); writing – original draft (equal); writing – review and editing (equal). **Surjit Sen:** Data curation (equal); investigation (equal); methodology (equal); writing – original draft (equal); writing – review and editing (equal). **Nilanjan Chakraborty:** Data curation (equal); investigation (equal); methodology (equal); writing – original draft (equal); writing – review and editing (equal). **Arghya Naskar:** Data curation (equal); investigation (equal); methodology (equal); writing – original draft (equal); writing – review and editing (equal). **Kairat Zhakipbekov:** Data curation (equal); investigation (equal); methodology (equal); supervision (equal); validation (equal); visualization (equal); writing – original draft (equal); writing – review and editing (equal). **Krishnendu Acharya:** Data curation (equal); investigation (equal); methodology (equal); supervision (equal); validation (equal); visualization (equal); writing – original draft (equal); writing – review and editing (equal). **Solomon Habtemariam:** Investigation (equal); methodology (equal); validation (equal); writing – original draft (equal); writing – review and editing (equal). **Dilek Arslan Ateşşahin:** Data curation (equal); investigation (equal); methodology (equal); writing – original draft (equal); writing – review and editing (equal). **Tamar Goloshvili:** Data curation (equal); investigation (equal); methodology (equal); writing – original draft (equal); writing – review and editing (equal). **Afaf Ahmed Aldahish:** Data curation (equal); investigation (equal); methodology (equal); validation (equal); visualization (equal); writing – original draft (equal); writing – review and editing (equal). **Javad Sharifi‐Rad:** Conceptualization (lead); data curation (equal); investigation (equal); methodology (equal); project administration (equal); supervision (equal); validation (equal); visualization (equal); writing – original draft (equal); writing – review and editing (equal). **Daniela Calina:** Data curation (equal); investigation (equal); methodology (equal); project administration (equal); supervision (equal); validation (equal); visualization (equal); writing – original draft (equal); writing – review and editing (equal).

## FUNDING INFORMATION

No funding received.

## CONFLICT OF INTEREST STATEMENT

None.

## Data Availability

The datasets generated during the current review are available from the corresponding author upon reasonable request.
